# Real world data on cardiometabolic diseases in U.S. adults during the SARS-CoV-2 pandemic: a decentralized registry study

**DOI:** 10.1186/s12933-022-01462-3

**Published:** 2022-02-14

**Authors:** Parth Shah, Kim Magee, Kiara H. Buccellato, McKenna Ismond, Jalisa Watson

**Affiliations:** ObvioHealth, 3452 Lake Lynda Dr., Bld. 100 Ste 151, Orlando, FL 32832 USA

**Keywords:** SARS-CoV-2, Diabetes, Cardiovascular, Metabolism, Insulin resistance, Angiotensin

## Abstract

**Background:**

Pre-existing cardiometabolic comorbidities place SARS-CoV-2 positive patients at a greater risk for poorer clinical course and mortality than those without it. We aimed to analyze real-world registry data focused primarily on participants with cardiometabolic diseases (CMD), which were remotely obtained via a digital platform.

**Methods:**

Participants were divided into two groups: CMD or no cardiometabolic disease (non-CMD). They were evaluated based on their medical history, current medications/supplements, COVID-19 status, demographics, and baseline characteristics. The frequency of medications/supplements for CMD were compared using relative risks and 95% confidence intervals. The WHO (Five) Well-Being Index (WHO-5) were collected monthly for 6 months to assess psychological well-being which included cheerfulness, calmness, vigor, rest, and engagement with daily activities of interest.

**Results:**

The 791 enrollees represented 49 U.S. states. The CMD group had significantly higher (p < 0.0001) BMI (mean + 3.04 kg/m^2^) and age (mean + 9.15 years) compared to non-CMD group. In the CMD group, participants who tested positive for COVID-19 had lower (p < 0.0001) well-being scores than those without COVID-19. For the 274 participants on CMD medications/supplements, there was no statistical difference in risk of COVID-19 contracture based on medication/supplement type; however, all six participants who were not being treated for CMD were COVID-19 positive (RR ~ 104). For 89 participants who were on treatment for diabetes or insulin resistance, there was a 90% reduced risk of COVID-19 incidence (p = 0.0187).

**Conclusion:**

The well-being score of the CMD group was dependent on whether they tested positive for COVID-19. Type of CMD treatment did not impact COVID-19 status, but absence of treatment significantly increased COVID-19 incidence. With respect to SARS-CoV-2, our analysis supports continued use of the statins, ACE-I, ARBs, and diabetes medications in CMD patients.

*Trial registration*: ClinicalTrials.gov Identifier: NCT04348942.

## Introduction

One third of the U.S. population suffers from metabolic syndrome [1] with prediabetes prevalence of 1 in 3 [2], diabetes impacting 34.2 million (10.5%) [3], and cardiovascular diseases being the number one cause of mortality in U.S. [4]. Pre-existing cardiometabolic comorbidities place SARS-CoV-2 positive patients at a clinically significant greater risk for worse clinical course and mortality [5]. Furthermore, COVID-19 hospitalizations in New York, the outbreak epicenter of the U.S., included largely patients with obesity (42%), diabetes (34%) and hypertension (57%) [5]. COVID-19 mortality and clinical severity are also significantly higher in African American and Hispanic minorities [6], in male patients, and those who are ≥ 65 years of age [7, 8].

The route of pathogenesis of SARS-CoV-2 plays an important role in its connection with metabolic diseases. The protease, furin, expressed at high levels in diabetics, allows for rapid activation of S protein which binds to angiotensin-converting enzyme-2 (ACE-2) receptors on cell surface, increasing the risk for SARS-CoV-2 infection [9, 10]. Patient body mass index (BMI) has shown a linear correlation with COVID-19 severity [11]. A greater proportion of adipose tissue results in elevated pro-inflammatory macrophages and CRP, leading to systemic meta-inflammation [5]. Pre-existing meta-inflammation, combined with the body’s inflammatory response to COVID-19, may coalesce to create a more clinically severe presentation of COVID-19 and higher risk of mortality [5].

COVID-19 cardiac damage may be permanent even following recovery. Cardiac injury secondary to SARS-CoV-2 can be indicated by elevated levels of the cardiac enzyme troponin, which can present in more than half of those with pre-existing heart conditions and 20% of all hospitalized patients [12]. Increased troponin is associated with a higher risk of cardioembolic stroke or embolic stroke of unknown origin [13]. COVID-19 derived cardiac damage is hypothesized as a cause of the high rate of stroke and stroke-related fatalities among COVID-19 patients [14]. Furthermore, SARS-CoV-2 infection induces a state of hypercoagulability due to its pro-inflammatory effect increasing the risk of thromboembolic events such as transient ischemic attack (TIA), stroke, and pulmonary embolism [15, 16]. In early stages of COVID-19, three to fourfold rise D-dimer has been correlated with poor prognosis and may be triggered by underlying conditions such as diabetes, cancer, stroke, and pregnancy [17]. Consequently, anti-coagulant use has been suggested in COVID-19 positive diabetics [18]. At the time of diagnosis of COVID-19 in patients with cardiometabolic diseases, prior use of oral anti-coagulants compared to Vitamin K antagonists demonstrated lower risk of arterial or venous thrombosis without increasing the risk of bleeding [19]. First-line treatment for stage I hypertension include angiotensin receptor blockers (ARBs) and angiotensin converting enzyme inhibitors (ACE-I) [20]. Recent evidence suggests that despite the involvement of ACE-2 in cellular entry of SARS-CoV-2 [21], ARBs and ACE-I class of medications are beneficial for those with COVID-19 [22].

The objective of this study was to analyze real-world registry data obtained remotely from participants via a digital platform (decentralized approach) across United States during the SARS-CoV-2 pandemic. Demographics, baseline characteristics, medical history, classes of medications/supplements consumed, psychological well-being scores, COVID-19 status, and COVID-19 associated ER visits or hospitalizations were analyzed to gain clinically meaningful insights. This report is focused primarily on participants with cardiometabolic diseases identified during the larger study.

## Methods

ClinicalTrials.gov Identifier: NCT04348942.

### Design

This study was approved by the institutional review board (IRB)—Integreview/Advarra—and all participants were consented prior to completing the study tasks. This prospective longitudinal registry study utilized a decentralized approach to track SARS-CoV-2 exposure and diagnosis in the general population. Adults (≥ 18 years) living throughout the United States with reliable access to a smartphone with internet capabilities were invited to participate during the enrollment period of May–September 2020. The enrollment was independent of any restricting criteria such as geographic location (within U.S.), gender, race, ethnicity, co-morbidities, SARS-CoV-2 status and course, and other characteristics. The study participation period was 6 months without any monetary compensation for participating. The decentralized approach deployed the ObvioHealth (OH) app and portal to connect the remote clinical site team with participants. The OH app permitted fully remote completion of all study tasks with automated notifications/reminders, and interactions with the clinical site team throughout the entire participant journey.

### Measures

All questionnaires were completed by the participants via the OH app. Baseline questionnaires or forms included: basic demographic information (age, gender, race, ethnicity, height, weight, geographic location); smoking status; medical history and current medications; recent exposure to a person known to have COVID-19 or recent experience that may produce higher than average risk of COVID-19; prior SARS-CoV-2 assessment(s) (i.e., laboratory testing and/or medical visit) and subsequent determination by the healthcare professional.

The observational period assessments included: daily reports of current wellness and COVID-19-like symptoms, as applicable; a monthly psychological well-being questionnaire; changes in medications, as applicable; reports of any emergency room visits or hospitalization, as applicable; and reports of the results of any SARS-CoV-2 tests, as applicable.

Responses to the WHO (Five) Well-Being Index (WHO-5) were collected monthly for 6 months to assess psychological well-being which included cheerfulness, calmness, vigor, rest, and engagement with daily activities of interest [23]. The WHO-5 captured the participant’s subjective psychological well-being by use of five questions with a 6-point Likert scale. The raw score was calculated by totaling the values (1–5) of the five answers. The total raw score ranging from 0 to 25 was multiplied by 4 and reported on a scale of 0 to 100, 0 representing worst possible and 100 representing best possible score.

Our cardiometabolic disease assessment focused on participants with conditions such as insulin resistance, diabetes mellitus, hypertension, hyperlipidemia, obesity, coronary heart disease, stroke, TIA, hypothyroidism, and thrombophilia. Baseline characteristics and reports during the observational period were analyzed for valuable clinical insights.

### Statistical analysis

For statistical analysis, RStudio Version 1.4.1103 was used. Participants were initially divided into two groups based on medical history of either having cardiometabolic disease (CMD) or no cardiometabolic disease (non-CMD). Participants were categorized based on their medical history and current medications/supplements as well as COVID-19 status. The COVID-19 status was determined based on the PCR test result. Demographic and baseline characteristics of the groups were compared using Wilcoxon Rank Sum tests. Statistical significance was defined at 5%. The frequency of medications/supplements for cardiometabolic disease were compared using relative risks and 95% confidence intervals.

## Results

### Enrollment distribution

**Fig. 1:**
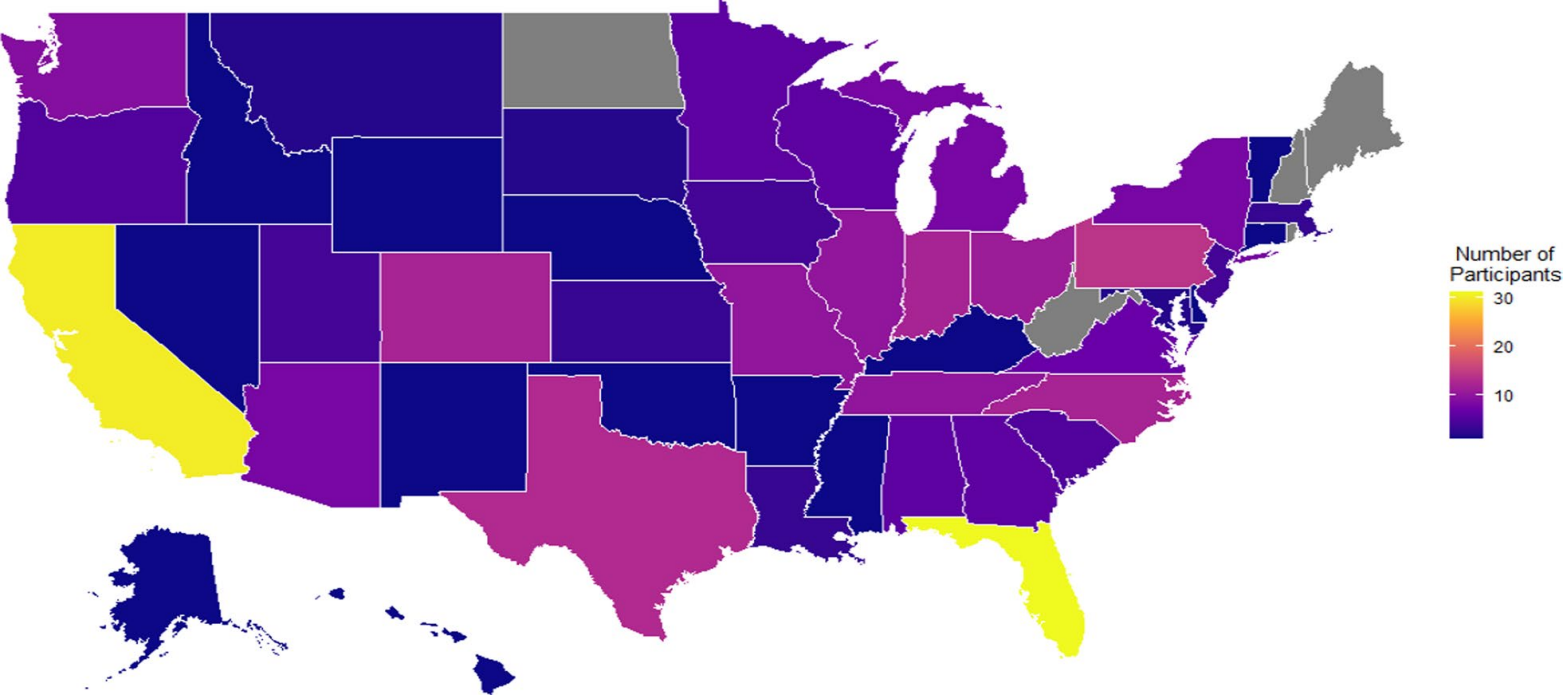
Distribution of CMD participants by state

The 791 enrollees represented 49 states, one state (New Hampshire) had no enrollees. Participants were initially divided into two groups: participants who had a history of either cardiometabolic disease (CMD; n = 280) or those that did not have a cardiometabolic disease (non-CMD; n = 511). As illustrated in Fig. 1, CMD participants resided in 45 states (5 states had no CMD participants). States shaded in grey were those not represented in the CMD group. When comparing enrollment distribution for 791 enrollees to the CMD participants, except for the four states where there were no CMD participants, the participant distribution was approximately parallel.

### Baseline characteristics and demographics

Baseline characteristics were not normally distributed and were assessed using the non-parametric Wilcoxon Rank Sum test. As illustrated in Table 1 and as anticipated, there was a statistically significant difference in the BMI, weight, and age of CMD participants compared to non-CMD participants, with CMD participants having higher values for each of these categories (p < 0.001 for each). In the CMD group, mean BMI (p = 0.369), Weight (p = 0.389), and Age (p = 0.398) were compared using a Wilcoxon Rank Sum test. No differences in these baseline characteristics were found between CMD COVID-19 positive and negative participants.

**Table 1:** Baseline characteristics by CMD status

	CMD	Non-CMD	p-value
	n = 280	n = 511	
	Mean (SD)	Mean (SD)	
BMI	31.91 (7.889) kg/m^2^	28.89 (7.289) kg/m^2^	< 0.001
Weight	201.00 (52.593) lbs	184.00 (50.349) lbs	< 0.001
Age	48.15 (13.932) years	39.00 (11.872) years	< 0.001

**Fig. 2:**
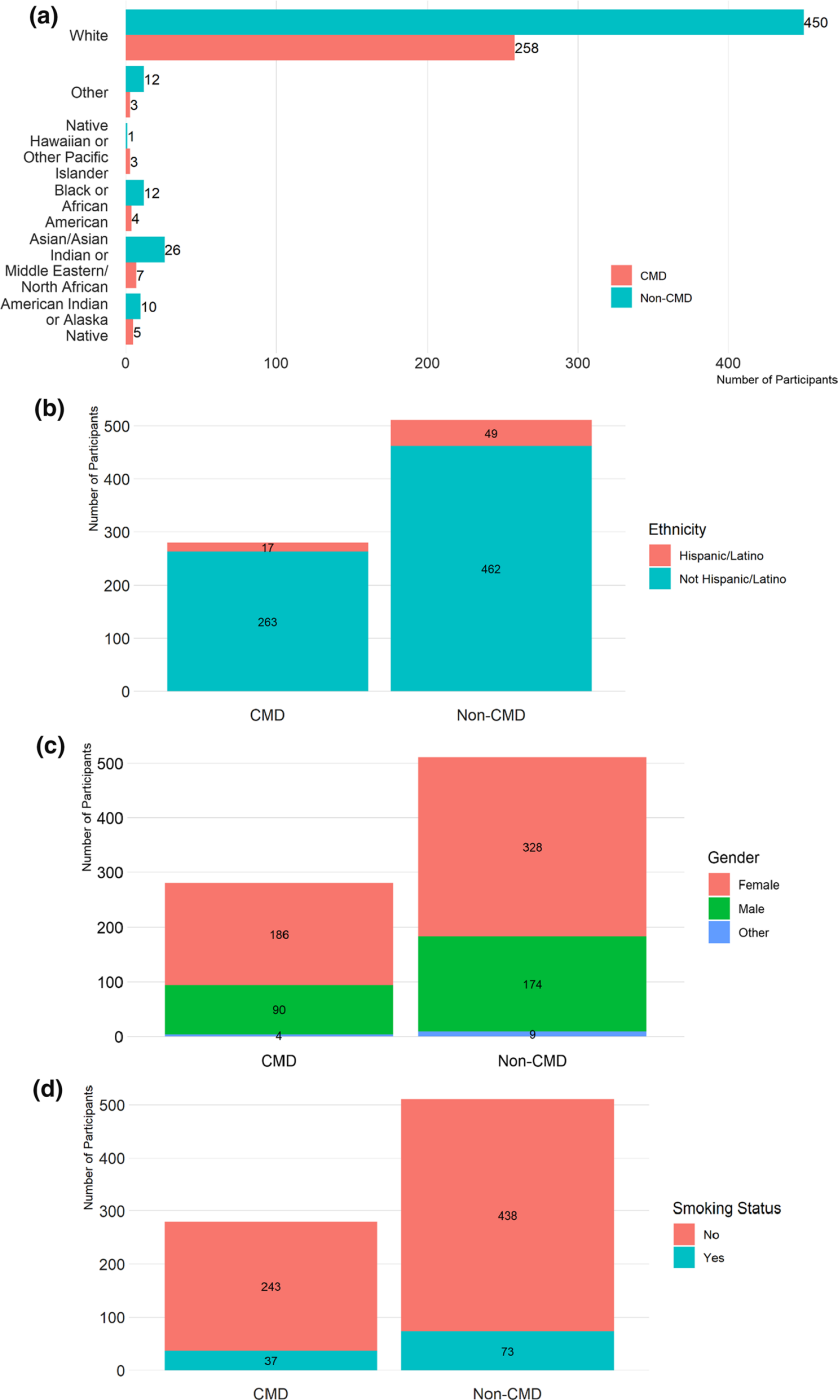
**a** Number of participants in CMD and Non-CMD groups by race. **b** Number of participants in CMD and Non-CMD groups by ethnicity. **c** Number of participants in CMD and Non-CMD groups by gender. **d** Number of participants in CMD and Non-CMD groups by smoking status

The demographics and smoking status are shown in (Fig. 2a–d). There were total of 280 CMD participants of which 92.1% were white and 66.4% female. Of the 511 non-CMD participants, 88.1% were white and 64.2% female. Overall, race and gender were not significantly different between CMD and non-CMD groups. The Hispanic/Latino, and smokers and non-smokers were approximately evenly distributed between CMD and non-CMD groups (Fig. 2a–d). Within the CMD group, there were 29 COVID-19 positive participants with 89.7% white and 75.9% female. Examining the remaining 251 COVID-19 negative CMD participants, 92.4% were white and 65.3% female. Race and gender were not significantly different within the CMD group based on COVID-19 status.

### Well-being scores

**Fig. 3:**
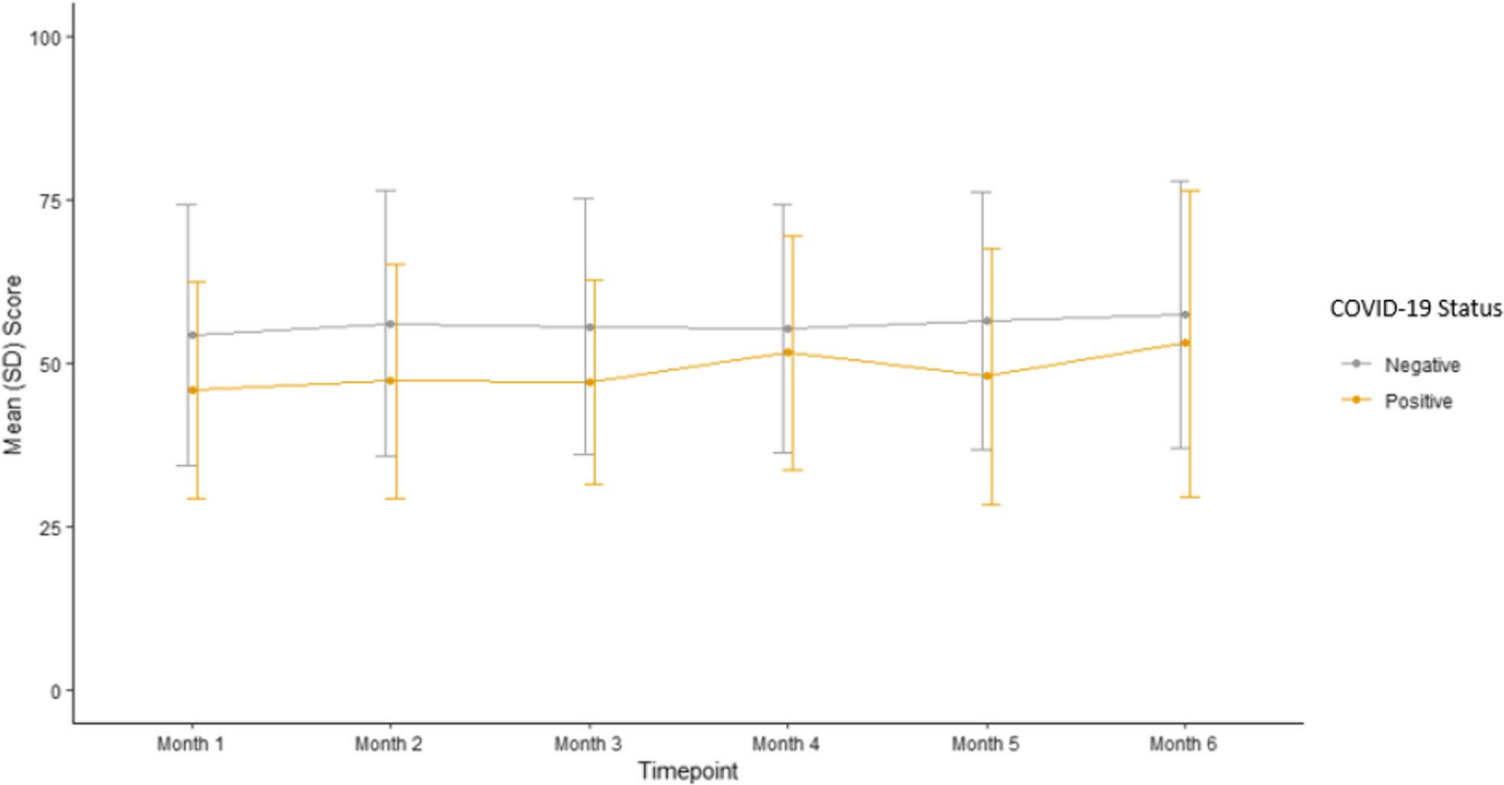
Mean wellbeing scores of CMD group by COVID-19 status per WHO-5 measure timepoints

**Fig. 4:**
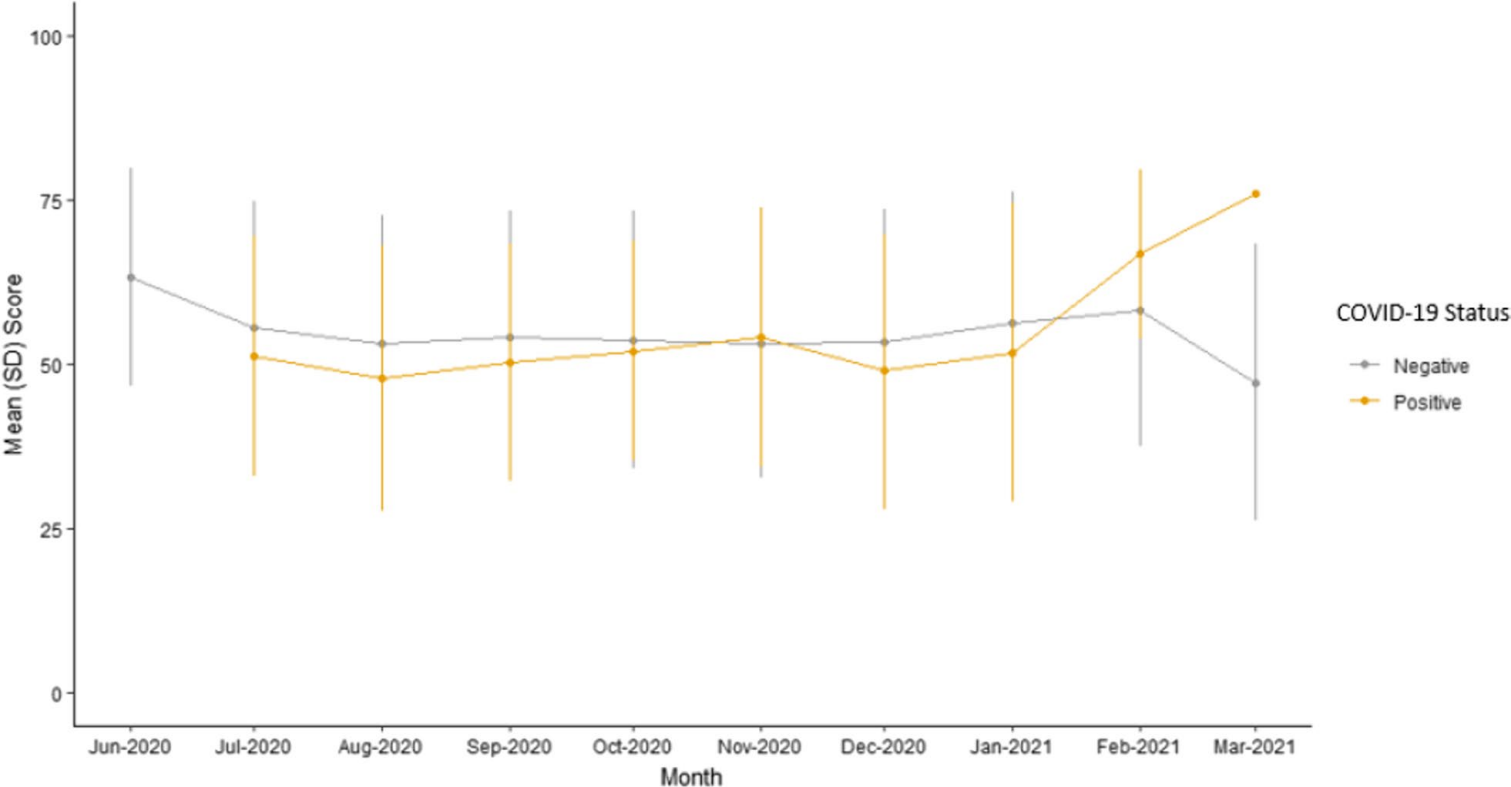
Mean wellbeing scores of CMD group by COVID-19 status over calendar time

Well-being scores were plotted graphically. Using a non-parametric Wilcoxon Rank Sum, no significant difference in total well-being scores was noted between CMD and non-CMD groups. Figure 3 displays the mean and standard deviation of well-being scores over each of the WHO-5 measure timepoints till the study exit for just CMD group based on positive or negative COVID-19 status. There is a statistically significant (p < 0.001) difference between the well-being scores of participants within the CMD group just based on their COVID-19 status. In the CMD group, participants who tested positive for COVID-19 (n = 23) had lower well-being scores (Month 1 average 45.8, Month 6 average 53) than those who were negative for COVID-19 (n = 163) (Month 1 average 54.3, Month 6 average 57.4). As illustrated in Fig. 4, mean well-being scores were also plotted by calendar month for CMD participants grouped by COVID-19 status. In this analysis, there was no significant statistical difference between the groups in any given calendar month.

### Cardiometabolic disease medications and supplements

CMD medications and supplements were classified into 32 different groups for examination. Table 2 shows the sub-categorized CMD medications and supplements with frequency ≥ 5%, Relative Risk and 95% Confidence Interval. The 14 treatment categories on the Table 2 include: Angiotensin Converting Enzyme Inhibitor, anti-coagulant, anti-platelet, Angiotensin Receptor Blocker, Beta-blocker, calcium channel blocker, non-statin hyperlipidemia treatment, diuretic, insulin, insulin sensitizer, statin, sulfonylurea, thyroid treatment, or no treatment.

**Table 2:** Frequency breakdown of category of CMD participants by COVID-19 status

Category	Positive		Negative		Total	Relative risk (95% CI)
	N	%	N	%		
Angiotensin converting enzyme inhibitor	6	20.69	44	17.53	50	1.18 (0.551, 2.527)
Anti-coagulant	2	6.9	5	1.99	7	3.46 (0.703, 17.048)
Anti-platelet	4	13.79	27	10.76	31	1.28 (0.483, 3.407)
Angiotensin receptor blocker	3	10.34	35	13.94	38	0.74 (0.243, 2.262)
Beta-blocker	5	17.24	52	20.72	57	0.83 (0.362, 1.915)
Calcium channel blockers	3	10.34	35	13.94	38	0.74 (0.243, 2.262)
Non-statin hyperlipidemia treatment	5	17.24	33	13.15	38	1.31 (0.556, 3.094)
Diuretic	2	6.9	43	17.13	45	0.4 (0.103, 1.576)
Insulin	0	0	13	5.18	13	0.33 (0.02, 5.465)
Insulin sensitizer	1	3.45	56	22.31	57	0.15 (0.022, 1.075)
Statin	7	24.14	66	26.29	73	0.92 (0.466, 1.808)
Sulfonylurea	0	0	15	5.98	15	0.29 (0.018, 4.703)
Thyroid medication	8	27.59	65	25.9	73	1.07 (0.57, 1.992)
No treatment	6	20.69	0	0	6	103.86 (5.952, 1812.352)^a^

Participants may appear in multiple categories

^a^Denotes statistically significant risk

For all participants who were on CMD medications/supplements, there was no statistical difference in the risk of COVID-19 contracture based on the medication/supplement type. However, a post hoc analysis of 89 participants who were on treatment for diabetes or insulin resistance had only one participant contracting COVID-19, indicating a 90% reduced risk of COVID-19 contracture (p = 0.0187) in this treated group. Six participants were COVID-19 positive and not on any treatment for a cardiometabolic disease. Analysis showed not taking CMD medications increased the risk of being COVID-19 positive by ~ 104-fold compared to those who were receiving treatment for CMD conditions. The participants not taking any CMD medications had a mean age of 36.3 years. Of the 6 participants with CMD who indicated they had not taken any treatment, one was only overweight, and 5 reported BMI > 30 kg/m^2^, and had conditions including hypercholesterolemia or diabetes, or had a previous history of gestational hypertension or gestational diabetes.

## Discussion

To our knowledge, this is the first decentralized real-world data registry study within the general United States adult population during the SARS-CoV-2 pandemic. The decentralized approach in this registry study allowed for enrollment of 791 participants across 49 U.S. states with those in CMD group representing 45 states (Fig. 1). This was likely due to recruitment being targeted to general U.S. adult population, and non-restrictive based on gender, race, ethnicity, COVID-19 status and course and other limiting parameters. The site team and participants were able to remotely interact and complete all study tasks through the digital platform, which was crucial to uninterrupted study conduct during the COVID-19 pandemic. Considering that there was no monetary reward for participation in this study, the altruistic response by the participants to help the scientific community better understand SARS-CoV-2 is applaudable. In this evaluation of our enrolled population, we focused on analyzing those participants with cardiometabolic diseases. This included conditions such as insulin resistance, diabetes mellitus, hypertension, hyperlipidemia, obesity, coronary heart disease, stroke, TIA, hypothyroidism, and thrombophilia.

In addition to considering the clinically diagnosed CMD co-morbidities, the baseline factors of higher BMI and age serve as an additional risk factor for SARS-CoV-2 infection. When comparing the CMD group to non-CMD group (Table 1), as expected, those in the CMD group had significantly higher weight (mean + 17 lbs), BMI (mean + 3.04 kg/m^2^) and were older (mean + 9.15 years). There was no significant difference in these parameters when comparing CMD cases based on COVID-19 status within the CMD group. There were 29 (10.4%) COVID-19 positive cases in CMD group versus 22 (5%) COVID-19 positive cases in non-CMD group, making it plausible that the BMI and age have an additive effect with the medically diagnosed CMD co-morbidities in incidence of SARS-CoV-2 infection. No participants in our study reported being hospitalized due to COVID-19; however, other reports from patients hospitalized for COVID-19 suggest a poorer clinical course and outcome for patients with higher BMI and age. Reports show for BMI ≥ 30 kg/m^2^, the risk of critical COVID-19 is 2.35-fold and its related mortality is 2.68-fold compared to < 30 kg/m^2^ group [11]. Consequently, critical COVID-19 and its mortality risk were even higher when age > 60 years was added to obesity with 3.11 and 3.93, respectively [11].

There was no significant difference between the CMD and non-CMD group based on race and gender, overall, ~ 90% of our study population was white and ~ 65% were women (Fig. 2a, c). This is likely due to the recruitment not being targeted based on race or gender. Of the 29 COVID-19 positive cases in CMD group, 76% were women and 90% of participants were white, which may be explained by a larger study participation of women and participants who identified themselves as white. The current literature shows no significant differences in COVID-19 incidence based on gender [24], but globally men tend to display a poorer clinical course to COVID-19 infection [8, 25]. Considering the racial/ethnic disparities, a study found Blacks/African-Americans had disproportionately high likelihood (OR = 2.6) of COVID-19 contracture [26]. Reports from New York suggested that mortality rates for African Americans and Latinos were two times higher than Whites and Asians [6]. However, in our CMD group there were only four Blacks/African Americans who were all on treatment and none were COVID-19 positive. The smokers (~ 14%) and non-smokers (~ 86%) were evenly distributed between the CMD and Non-CMD groups. Studies from hospitalized patients show that patients with COPD and ongoing smoking have a poor clinical course and prognosis [27].

There were 32 medication/supplement groups which 274 participants were using to treat their cardiometabolic diseases. In Table 2, the medications/supplements that CMD participants used with frequency ≥ 5% are shown. Overall, our findings show no statistically significant difference in COVID-19 status depending on the medication/supplement taken. All six participants who did not use any medications/supplements to treat their cardiometabolic diseases were COVID-19 positive. Consequently, although there is no statistical significance between the COVID-19 status based on the medication/supplement used, a grouped-analysis on those who were on any treatment for diabetes or insulin resistance revealed that the treated participants had a 90% reduced risk of COVID-19 contracture (p = 0.0187) which is of clinical relevance in supporting treatment of these conditions to reduce the incidence of SARS-CoV-2. In diabetics, impaired phagocytic cellular capabilities, and high levels of furin expression allow rapid activation of S protein leading to greater risk for SARS-CoV-2 infection [9, 10]. Uncontrolled blood glucose can lead to increased rate of SARS-CoV-2 related hospitalizations with intensive care unit admission and mortality while optimum control may improve the outcomes [26, 28]. A study in 432 diabetics found that the risk of pneumonia was markedly higher in Dipeptidyl peptidase-4 (DPP-4) inhibitor users, but no association was found between oral anti-diabetics use and COVID-19 related mortality [29]. Interestingly, a retrospective study looking at 25,326 participants tested for COVID-19 at a single center found that while diabetes significantly increased mortality odds (OR = 3.62), taking metformin independently and significantly reduced the odds, OR = 0.33 [26]. Supportively, hospitalization was not reported for our one COVID-19 positive participant who was on an insulin sensitizer.

There were only six participants who reported a non-treated CMD condition, and all were COVID-19 positive. Their risk of SARS-CoV-2 contracture was significant: ~ 104 times that of the treated group. The average age of this group was 36.3 years, and all were non-smokers. The group included five females, two with Class III obesity (BMI > 40), one with Class II obesity (BMI 35–39.9), two with Class I obesity (BMI 30–34.9), and one was merely overweight. In these six individuals, non-treated CMD co-morbidities included hypercholesterolemia or diabetes, or history of gestational hypertension or gestational diabetes. Per literature, obesity and diabetes coexistence contribute to worse prognosis of SARS-CoV-2 infection, but there is no clear evidence of higher predisposition to the infection [30]. The gestational diabetes mellitus (GDM) can be treated as a marker of insulin resistance leading to diabetes mellitus II long-term in approximately 50% of women [31, 32]. Per earlier discussion of the literature, optimum treatment of diabetes and insulin resistance may have protective effects against SARS-CoV-2. In 4,103 COVID-19 patients in New York, age was the strongest risk predictor of hospitalization followed by BMI > 40 kg/m^2^ [33]. While in 124 patients in France, BMI ≥ 35 kg/m^2^ was independently associated with requiring invasive mechanical ventilation [34, 35].

The SARS-CoV-2 virus binds and enters via the angiotensin converting enzyme 2 [36]. There is some debate in the literature whether medications such as ACE-I, ARBs, and statins influence the ACE-2 activity leading to higher incidence and poorer clinical outcomes, including mortality [24] or lead to protective effects against SARS-CoV-2 [21, 22, 37, 38]. Studies have shown that statins are correlated with significantly lower mortality in patients with COVID-19, thought to be due in part to blood-lipid reduction which may interfere with viral entry into the lipid membrane of the host cell as well as the anti-inflammatory effects of ACE-2 upregulation [37]. Furthermore, in hospitalized COVID-19 patients, particularly diabetics, statin use has been shown to significantly reduce mortality [39]. Our study found no significant difference in COVID-19 incidence based on the use of ACE-I, ARBs or statins. Furthermore, none of our participants reported being hospitalized for SARS-CoV-2. Overall, our study, in alignment with majority of studies, supports the continued use of the statins, ACE-I, and ARBs in CMD patients with respect to the SARS-CoV-2 infection.

The COVID-19 pandemic created a disturbance in daily lives of many in the United States. The impact included social isolation, working from home, anxiety of illness, etc. which likely affected the population’s psychological well-being. The WHO-5 index evaluated the psychological well-being of participants over a 6-month study participation period ranging from June 2020 to March 2021 during the COVID-19 pandemic. When comparing the CMD versus non-CMD group, there was no significant difference in change in well-being scores over the 6-month study participation period. However, within the CMD group, there was a significant difference during the 6-month WHO-5 assessments in mean scores based on the COVID-19 status with COVID-19 positive group average scores remaining lower. Furthermore, considering the months June 2020 to Mar 2021 within the CMD group and comparing well-being scores based on the COVID-19 status, we found no significant difference. Overall, the results of WHO-5 analysis show that in participants with cardiometabolic diseases, COVID-19 status was most likely an independent factor influencing participant’s quality of life during the pandemic months.

The limitations of this real-world study include: enrollment of general U.S. adult population which was independent of restricting criteria such as geographic location (within U.S.), gender, race, co-morbidities, SARS-CoV-2 status and course, and other characteristics; limited sample sizes based on participant categorization into treatment classes and SARS-CoV-2 status supporting a need for larger treatment focused studies to further validate the conclusions; and lack of data collection on socio-economic class, education, and daily activities.

## Conclusion

Using the decentralized approach in this registry study, real world data was gathered remotely from U.S. adults during the SARS-CoV-2 pandemic. The CMD group had higher BMI and age compared to non-CMD, and among the CMD group, the well-being score was dependent on COVID-19 status (i.e., the infection positive cases displayed a lower well-being score). The type of cardiometabolic disease treatment did not impact COVID-19 status, but non-treatment significantly increased COVID-19 incidence. A grouped-analysis on those who were receiving any treatment for diabetes or insulin resistance revealed that the treated participants had a 90% reduced risk of COVID-19 incidence (p = 0.0187). This data supports treatment compliance in these conditions to reduce the incidence of SARS-CoV-2. Our study further supports the continued use of the statins, ACE-I, and ARBs in CMD patients with respect to the SARS-CoV-2 infection.

## Data Availability

All data were captured in the ObvioHealth platform and all study materials were provided by ObvioHealth.

## Abbreviations

ACE-2: Angiotensin-converting enzyme-2
ACE-I: Angiotensin converting enzyme inhibitor
ARB: Angiotensin receptor blocker
BMI: Body mass index
CMD: Cardiometabolic diseases
COVID-19: Coronavirus disease 2019
DPP-4: Dipeptidyl peptidase-4
GDM: Gestational diabetes mellitus
OH: ObvioHealth
SARS-CoV-2: Severe acute respiratory syndrome coronavirus 2
TIA: Transient ischemic attack
U.S.: United States of America
WHO-5: World Health Organization (five) well being index
